# Anti-factor Xa Activity Is Not Associated With Venous Thromboembolism in Critically Ill Patients Receiving Enoxaparin for Thromboprophylaxis: A Retrospective Observational Study

**DOI:** 10.3389/fmed.2022.888451

**Published:** 2022-04-29

**Authors:** Christoph Dibiasi, Johannes Gratz, Marion Wiegele, Andreas Baierl, Eva Schaden

**Affiliations:** ^1^Department of Anaesthesia, Intensive Care Medicine and Pain Medicine, Medical University of Vienna, Vienna, Austria; ^2^Ludwig Boltzmann Institute for Digital Health and Patient Safety, Vienna, Austria; ^3^Department of Statistic and Operations Research, University of Vienna, Vienna, Austria

**Keywords:** anti-factor Xa activity, low molecular weight heparin, thromboprophylaxis, venous thromboembolism, critical illness, anticoagulation

## Abstract

**Background:**

Anti-factor Xa activity has been suggested as a surrogate parameter for judging the effectiveness of pharmacological thromboprophylaxis with low molecular weight heparins in critically ill patients. However, this practice is not supported by evidence associating low anti-factor Xa activity with venous thromboembolism.

**Methods:**

We performed a retrospective observational study including 1,352 critically ill patients admitted to 6 intensive care units of the Medical University of Vienna, Austria between 01/2015 and 12/2018. Included patients received prophylactically dosed enoxaparin (≤100 IU/kg body weight per day). We analyzed median peak, 12-h trough and 24-h trough anti-factor Xa activity per patient and compared anti-factor Xa activity between patients without vs. with venous thromboembolic events.

**Results:**

19 patients (1.4%) developed a total of 22 venous thromboembolic events. We did not observe a difference of median (IQR) anti-factor Xa activity between patients without venous thromboembolism [peak 0.22 IU/mL (0.14–0.32); 12-h trough 0.1 IU/mL (<0.1–0.17), 24-h trough < 0.1 IU/mL (<0.1– <0.1)] vs. patients with venous thromboembolism [peak 0.33 IU/mL (0.14–0.34); 12-h trough 0.12 IU/mL (<0.1–0.26); 24-h trough < 0.1 IU/mL (<0.1–<0.1)].

**Conclusion:**

Patients who developed venous thromboembolism had anti-factor Xa activities comparable to those who did not suffer from venous thromboembolism.

## Introduction

Venous thromboembolism (VTE), comprising deep vein thrombosis (DVT) and pulmonary embolism (PE), is a common complication in hospitalized patients. Critically ill patients have a particularly high risk of VTE, and reported incidences vary between 1.4 and 15% (1–4). According to current European guidelines, pharmacological VTE prophylaxis in patients admitted to an intensive care unit (ICU) should preferentially be conducted using low molecular weight heparins (LMWH) at a fixed dose (2). LMWH can be monitored using the anti-factor Xa activity (antiXa) assay, but target ranges have thus far only been established for therapeutic anticoagulation (5). In patients receiving LMWH for thromboprophylaxis, antiXa should only be monitored to exclude LMWH accumulation when severe renal insufficiency is present (2). The utility of antiXa in judging the efficacy of LMWH thromboprophylaxis remains controversial, and it is uncertain whether (compared to patients on normal wards) decreased antiXa levels regularly encountered in critically ill patients are associated with an increased risk of VTE (6, 7). Nevertheless, recent studies have suggested lower VTE rates using dose-adjusted thromboprophylactic LMWH regimes targeting arbitrarily defined antiXa thresholds (8, 9). The results of these studies are limited by their small sample sizes, heterogeneous target antiXa thresholds and the uncertainty of whether peak or trough antiXa should be obtained. We thus performed a large retrospective observational study describing antiXa of critically ill patients who received prophylactically dosed enoxaparin. In addition, we assessed the relationship between antiXa and the development of VTE.

## Materials and Methods

### Data Collection and Processing

This study was approved by the ethics committee of the Medical University of Vienna, Austria (reference number 1936/2019). The need to obtain informed consent was waived by the ethics committee due to the retrospective nature of this study. We screened the electronic health records of all patients admitted to six ICUs at the General Hospital of Vienna, a tertiary care center of the Medical University of Vienna, Austria, from 01/2015 to 12/2018 for eligibility. Patients were eligible if their age at the time of admission exceeded 18 years, the length of stay in the ICU exceeded 24 h and antiXa (calibrated for LMWH) was measured at least once during the ICU stay.

Data of eligible patients was exported from the IntelliSpace Critical Care and Anaesthesia patient data management system (Philips Austria GmbH, Vienna, Austria). Exported data included basic demographic data (age, weight, height, body mass index and primary diagnosis) at admission, all antiXa measured, and any antithrombotic medication administered during the ICU stay. In addition, we exported data on prothrombine time (Owren, reference range 24.6–32.7 s), activated partial thromboplastin time (reference range 27–41 s), fibrinogen concentration (Clauss, reference range 2–4 g L^–1^) and antithrombin III activity (reference range 80–120%). We collected data from all eligible patients with at least one valid antiXa. In the final analysis, we included patients who received prophylactic anticoagulation with enoxaparin either for the first 4 days after ICU admission or on 85% of overall ICU days. For this study, we defined prophylactic anticoagulation as a cumulative daily dose of ≤ 100 IU enoxaparin per kg actual body weight.

AntiXa was measured in the central clinical laboratory using the STA^®^-Liquid Anti-Xa assay (reference numbers 00311 and 00322, Diagnostica Stago, Asnières-sur-Seine, France) on a STA R Max 2 (Diagnostica Stago SAS, Asnières-sur-Seine, France). The detection range for this assay is 0.1–2.0 IU/mL. Measurement of antiXa was performed according to attending ICU clinicians. Clinical reasoning for obtaining antiXa was not documented. For each antiXa, we calculated the duration between the last documented enoxaparin administration and the time of measurement as documented by the lab report. Subsequently, we categorized each antiXa as either peak (interval 3–5 h), 12-h trough (interval 11–13 h) or 24-h trough (interval 23–25 h). We excluded all antiXa for which any of the following was true:

1. AntiXa was measured outside the ICU stay.
2. Oral or parenteral anticoagulants other than enoxaparin (rivaroxaban, dabigatran, apixaban, edoxaban, phenprocoumon, argatroban, and fondaparinux) were administered in the last 48 h before antiXa was determined.
3. Unfractionated heparin was administered on the same day as antiXa was measured.
4. Extracorporeal membrane oxygenation (ECMO) was instituted before the antiXa measurement.
5. AntiXa was measured after the patients had developed VTE.
6. No enoxaparin administration was documented before the antiXa measurement.
7. AntiXa could not be classified as peak, 12-h trough or 24-h trough, as described above.

### Venous Thromboembolism

We defined VTE as either (a) lower or upper extremity DVT without intravenous catheters at the same anatomic site or (b) PE. DVT was diagnosed by duplex sonography and PE was diagnosed using computed tomography. As recommended by current guidelines, routine ultrasound screening for DVT is not conducted at our institution (2).

To assess the occurrence of VTE in our patient cohort, we screened admission and discharge documents alongside daily clinical progress notes and searched for keywords indicating the presence of lower or upper extremity DVT, respectively, PE. The list of keywords was compiled after manually reviewing the records of 198 eligible patients from 01/2018 to 05/2018. Search strategy, keywords and detailed results are provided as Supplementary Material. We regarded patients without any positive matches during the search as not having developed VTE during their ICU stays. Clinical notes identified by automated screening were subsequently assessed manually by one investigator (CD). If VTE was documented in the patient notes, its type (lower extremity DVT, upper extremity DVT, PE), anatomic location and date of diagnosis were recorded. Patients with pre-existing VTE at ICU admission were excluded from the analysis.

### Statistical Analysis

Categorical variables are given as absolute and relative frequencies. Continuous variables are given as medians with first and third quartiles. Wilcoxon rank sum tests were used for comparisons between two groups. Fisher’s exact test and Pearson’s Chi-squared test were used for comparison of categorial variables. To account for multiple antiXa measurements per patient, we summarized antiXa by calculating median, minimum and maximum antiXa per patient. All statistical tests were two-sided, and *p*-values < 0.05 were considered statistically significant.

### Software

Available data was exported into comma-separated value files via the structured query language interface using Microsoft SQL Server Management Studio 17.9.1 (Microsoft, Redmond, Washington, United States of America). Data processing was conducted using R version 4.04 (R Foundation for Statistical Computing, Vienna, Austria).^1^ Clinical notes were searched using Python version 3.7.7 (Python Software Foundation, Beaverton, Oregon, United States) using the *re* regular expression library.

## Results

**FIGURE 1:**
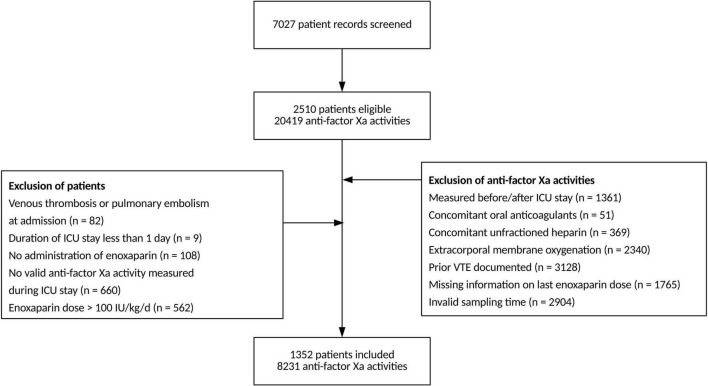
Strengthening the Reporting of Observational studies in Epidemiology (STROBE) flow chart.

We assessed electronic health records of 2,510 eligible patients and included 1,352 patients with a total of 8,231 antiXa in the final analysis (Figure 1). Table 1 presents the baseline patient data.

**TABLE 1:** Baseline patient data.

Measures	No thromboembolic complications *n* = 1333	Venous thromboembolism *n* = 19	*p*-value
Age (years)	64 (52–73)	61 (40–72)	0.30
Male gender (%)	828 (62)	10 (53)	0.40
Weight (kg)	78 (65–90)	76 (58–94)	0.99
Height (cm)	172 (165–179)	170 (165–178)	0.79
Body mass index (kg m^–2^)	26 (22–30)	22 (21–27)	0.14
Severe acute physiology score at admission (-)	52 (40–65)	58 (52–82)	0.009
ICU length of stay (days)	6 (2–13)	48 (18–84)	<0.001
ICU mortality (%)	98 (7.4)	6 (32)	0.002
**Patient groups**			0.648
Elective surgery (%)	619 (51)	10 (53)	
Emergency surgery/Trauma (%)	361 (30)	7 (37)	
Medical (%)	228 (19)	2 (11)	
**Comorbidities**			
Arterial hypertension (%)	594 (48)	8 (42)	0.59
Heart failure (%)	219 (18)	4 (21)	0.76
Diabetes mellitus (%)	226 (18)	5 (26)	0.37
Chronic obstructive pulmonary disease (%)	232 (19)	0 (0)	0.03
Malignant disease (%)	144 (12)	4 (21)	0.27
Chronic renal failure (%)	204 (17)	8 (42)	0.01
Liver cirrhosis (%)	94 (7.7)	2 (11)	0.65
**Laboratory coagulation parameters at admission**			
Activated partial thromboplastin time (s)	38 (34–44)	38 (36–48)	0.46
Prothrombin time (s)	33 (29–38)	37 (31–46)	0.039
Fibrinogen concentration (g l^–1^)	3.86 (2.89–5.17)	4.89 (2.86–5.77)	0.46
Antithrombin III activity (%)	78 (62–93)	72 (48–89)	0.25

*271 patients did not have height data available; 85 patients had no information on severe acute physiology score; 125 patients did not have information on surgical status and 105 patients had no information on comorbidities available. p-values were obtained using Pearson’s Chi-squared test (gender, comorbidities), Fisher’s exact test (for patient groups, ICU mortality) and Wilcoxon rank sum test (for all remaining variables).*

Nineteen patients (1.4%) developed a total of 22 VTE (seven lower extremity DVT, five upper extremity DVT and ten PE). A detailed description of patients developing VTE is given as Supplementary Material. The median time to diagnosis of VTE after ICU admission was 36 days (4–48). All patients received pharmacological thromboprophylaxis with subcutaneous enoxaparin, which was started at a median of 8 h (6–16) after ICU admission, with a median daily cumulative dose of 4000 IU (4000–4900) on a median of 100% (94–100) of ICU days. We found no differences between patients without thrombosis and patients who developed VTE regarding the duration until the start of pharmacological thromboprophylaxis [8 (6–16) vs. 8 h (6–24), *p* = 0.61], cumulative daily enoxaparin dose [4000 IU (4000–4400) vs. 4200 IU (4000–5600), *p* = 0.23] and the proportion of days with pharmacological thromboprophylaxis [100 (94–100) vs. 96% (90–100), *p* = 0.06].

Overall, the included patients had a median of 2 (1–5) peak, 2 (1–6) 12-h trough and 2 (1–5) 24-h trough antiXa values. The median number of peak antiXa measurements in the respective subgroups (patients without thrombosis and patients with VTE) were 2 (1–5) vs. 1 (1–2; *p* = 0.31). The median number of 12-h trough values was 2 (1–6) vs. 5 (3–10; *p* = 0.07) and the median number of 24-h trough values was 2 (1–5) vs. 1 (1–4; *p* = 0.31). Table 2 shows median, minimum and maximum antiXa at the patient level in comparison between patients without thrombosis and patients with VTE.

**TABLE 2:** Aggregated anti-factor Xa activity stratified by development of VTE.

Anti-factor Xa Level	No thromboembolic complications *n* = 1,333	Venous thromboembolism *n* = 19	*p*-value
*Median anti-factor Xa values (IU/mL)*			
Peak	0.22 (0.14–0.32)	0.33 (0.14–0.34)	0.29
12-h trough	0.1 (<0.1–0.17)	0.12 (<0.1–0.26)	0.37
24-h trough	<0.1 (<0.1–<0.1)	<0.1 (<0.1–<0.1)	0.32
*Minimum anti-factor Xa values (IU/mL)*			
Peak	0.16 (<0.1–0.24)	0.30 (0.09–0.33)	0.13
12-h trough	<0.1 (<0.1–0.14)	<0.1 (<0.1–0.2)	0.26
24-h trough	<0.1 (<0.1–<0.1)	<0.1 (<0.1–<0.1)	0.45
*Maximum anti-factor Xa values (IU/mL)*			
Peak	0.29 (0.16–0.41)	0.33 (0.25–0.37)	0.67
12-h trough	0.14 (<0.1–0.21)	0.16 (<0.1–0.4)	0.32
24-h trough	<0.1 (<0.1–0.12)	<0.1 (<0.1–<0.1)	0.19

*Median, minimum and maximum anti-factor Xa activities were calculated for each patient and summarized using median (interquartile range). p-values were obtained using Wilcoxon rank sum tests.*

In patients developing VTE, the most recent antiXa was obtained at a median of 2.5 days (1–25) prior to diagnosis of VTE. Median peak, 12 h and 24 trough antiXa prior to diagnosis of VTE were 0.27 IU/mL (0.1–0.3), 0.14 IU/mL (0–0.21) and 0 IU/mL (0–0).

Given that patients with VTE had a significantly higher severe acute physiology III (SAPS) score at admission, we performed a subgroup analysis and analyzed the relationship of antiXa with VTE in each of the four SAPS quarters. Median SAPS at admission in the first, second, third and fourth quarters was 35 (29–38), 46 (43–49), 58 (56–62), and 74 (69–81). The number of patients developing VTE in the respective groups was 3 of 317 (0.95%), 2 of 317 (0.63%), 6 of 317 (1.89%), and 8 of 316 (2.53%). For 85 patients, SAPS III at admission was missing and no patients developed VTE in this group. Median antiXa was not different between patients without VTE vs. patients who developed VTE in each of the four SAPS quarters. A detailed table is provided as Supplementary Material.

## Discussion

For this study, we analyzed a large cohort of critically ill patients and described antiXa obtained after prophylactic administration of enoxaparin. We also investigated the possible association between antiXa and the occurrence of VTE. Although 12 and 24-h trough antiXa were below thresholds that have been suggested to indicate effective thromboprophylaxis in previous studies, the incidence of VTE in our patient cohort was remarkably low. We did not identify a difference in peak, 12-h trough or 24-h trough antiXa between patients who did not develop venous thromboembolism and patients with VTE.

Throughout previous studies investigating weight-based or antiXa-guided LMWH dosing, considerable incongruity exists in which antiXa thresholds are regarded as indicating effective thromboprophylaxis. For instance, the combination of peak antiXa > 0.2 IU/mL and trough > 0.1 IU/mL was used in one study (10), whereas only trough levels between 0.1–0.2 IU/mL (9), peak levels > 0.2 IU/mL (8) or peak levels between 0.1–0.3 IU/mL (11), 0.2–0.5 IU/mL (12) or 0.3–0.5 IU/mL (13) were considered as targets by others. According to these studies, the patients included in the present study would have largely been classified as receiving inadequate pharmacological thromboprophylaxis. However, we observed a low VTE rate of 1.4%.

Compared to patients without thromboembolic complications, we found no differences in median, minimum or maximum antiXa in patients who developed VTE. This contrasts recent studies suggesting the measurement of antiXa to guide LMWH dosing for pharmacological thromboprophylaxis (14). The origins of antiXa-guided thromboprophylaxis are largely rooted in a study that found 12-h trough antiXa of at least 0.1 IU/mL to be associated with a significantly reduced VTE rate in non-critically ill patients (15). However, those findings lacked reproducibility in a similar study conducted 10 years later (16). Regarding critically ill patients, a recent systematic review found no association between antiXa and the occurrence of VTE (17). It reported high heterogeneity in-between studies, which may reflect poor study quality. Of the 18 included studies, only Malinoski et al. found an association between antiXa and the occurrence of VTE. The authors investigated 54 critically ill trauma patients and showed that 11 of 27 patients with a 12-h trough antiXa ≤ 0.1 IU/mL developed VTE compared to 3 of 27 patients with a trough antiXa > 0.1 IU/mL (7). The generalizability of these results may, however, be questionable because of the small sample size and routine screening for VTE, which may have inflated the reported VTE incidence. Similarly, Ko et al. who reported a lower VTE rate in critically ill injured patients receiving antiXa-guided thromboprophylaxis, employed routine ultrasound screening for the detection of VTE (9). In addition, the statistical significance of their findings was largely based on the differences in isolated distal DVT. However, our data support the results of various previous studies demonstrating that low antiXa is highly prevalent in critically ill patients but cannot be linked to VTE (18–21).

The low VTE rate of 1.4% found in this study supports recent studies reporting similar incidences (1, 22) but contradicts earlier studies that reported substantially higher rates of VTE in critically ill patients (4, 23). This can be explained by the early initiation of pharmacological thromboprophylaxis after a median duration of 8 h following ICU admission in our patient cohort, which has been shown to reduce VTE incidence (24–26). However, previous studies investigating antiXa-guided dosing of LMWH for thromboprophylaxis have reported delays of up to 7 days after ICU admission until thromboprophylaxis was started (3, 9, 11). In addition, enoxaparin was administered on nearly all days that patients were admitted to an ICU. Avoidance of missing LMWH doses is also known to reduce VTE incidence in critically ill patients (25). Another possible explanation for low VTE rates is that duplex ultrasound screening for DVT is not routinely performed at our institution. This follows current guidelines (2), given that the clinical impact of asymptomatic DVT is unclear (27). We also found that patients who developed VTE were significantly sicker at the time of ICU admission, as reflected by higher severe acute physiology scores and had a longer ICU stay alongside increased mortality. Thus, VTE might not necessarily represent failure of thromboprophylaxis but could rather be a manifestation of prolonged critical illness, as recently suggested (28).

Several limitations hinder the generalizability of our results and highlight the need for well conducted prospective trials. Most importantly, the retrospective nature of our study introduces a relevant risk of bias. Our assessment of the presence or absence of VTE relied on correct documentation by ICU physicians. It is thus possible that documented VTE were not captured by our search strategy or that VTE were not sufficiently documented by healthcare providers, both of which might cause an underestimation of true VTE incidence. Also, we did not assess VTE diagnosed after ICU discharge, which could possibly be dependent on thromboprophylaxis implemented during the ICU stay. As a result, our study is possibly underpowered due to the low VTE rate despite including a large cohort. We also did not conduct a power analysis for this study, but rather included all available patients confirming to inclusion, respectively exclusion criteria. Furthermore, we could not assess potential confounders, such as prior thromboembolic events or malignant disease in the patients’ past medical histories. However, we demonstrated that a low rate of clinical significant VTE can be achieved in the setting of highly effective prophylactic anticoagulation despite antiXa, that was lower than previously suggested “protective” levels.

In summary, we analyzed a cohort of 1,352 critically ill patients who received enoxaparin for pharmacological thromboprophylaxis according to current guidelines and found antiXa trough levels below thresholds often cited as being protective of VTE. Yet, we found a low VTE incidence of 1.4%. Patients who acquired VTE had similar peak and trough antiXa levels compared to those who did not suffer from thromboembolic complications. AntiXa thresholds protective of VTE still need to be determined for critically ill patients receiving prophylactically dosed LMWH.

## Data Availability Statement

The raw data supporting the conclusions of this article will be made available by the authors, without undue reservation.

## Ethics Statement

The studies involving human participants were reviewed and approved by Ethics Committee of the Medical University of Vienna. Written informed consent for participation was not required for this study in accordance with the national legislation and the institutional requirements.

## Author Contributions

CD and ES conceived and planned the study. CD collected the data, performed the initial data analysis, and drafted the first version of the article. CD and AB performed the final statistical analysis. CD, JG, MW, ES, and AB critically discussed and interpreted the data. JG, MW, AB, and ES critically revised the manuscript. All authors read and approved the final manuscript.

## Conflict of Interest

The authors declare that the research was conducted in the absence of any commercial or financial relationships that could be construed as a potential conflict of interest.

## Publisher’s Note

All claims expressed in this article are solely those of the authors and do not necessarily represent those of their affiliated organizations, or those of the publisher, the editors and the reviewers. Any product that may be evaluated in this article, or claim that may be made by its manufacturer, is not guaranteed or endorsed by the publisher.

## Footnotes

1
https://www.r-project.org

## References

[1] Viarasilpa T Panyavachiraporn N Marashi SM Van Harn M Kowalski RG Mayer SA. Prediction of symptomatic venous thromboembolism in critically Ill patients: the ICU-venous thromboembolism score. *Crit Care Med.* (2020) 48:e470. 10.1097/CCM.0000000000004306 32187076

[2] Duranteau J Taccone FS Verhamme P Ageno W ESA VTE Guidelines Task Force. European guidelines on perioperative venous thromboembolism prophylaxis: intensive care. *Eur J Anaesthesiol.* (2018) 35:142–6. 10.1097/EJA.0000000000000707 29112545

[3] Lim W Meade M Lauzier F Zarychanski R Mehta S Lamontagne F Failure of anticoagulant thromboprophylaxis: risk factors in medical-surgical critically Ill patients. *Crit Care Med.* (2015) 43:401–10. 10.1097/CCM.0000000000000713 25474533

[4] Shorr AF Williams MD. Venous thromboembolism in critically ill patients: observations from a randomized trial in sepsis. *Thromb Haemost.* (2009) 101:139–44. 10.1160/TH08-07-0468 19132200

[5] Garcia DA Baglin TP Weitz JI Samama MM. Parenteral anticoagulants: antithrombotic therapy and prevention of thrombosis, 9th ed: American college of chest physicians evidence-based clinical practice guidelines. *Chest.* (2012) 141:e24S–43S. 10.1378/chest.11-2291 22315264 PMC3278070

[6] Priglinger U Delle Karth G Geppert A Joukhadar C Graf S Berger R Prophylactic anticoagulation with enoxaparin: is the subcutaneous route appropriate in the critically ill? *Crit Care Med.* (2003) 31:1405–9. 10.1097/01.CCM.0000059725.60509.A0 12771610

[7] Malinoski D Jafari F Ewing T Ardary C Conniff H Baje M Standard prophylactic enoxaparin dosing leads to inadequate anti-Xa levels and increased deep venous thrombosis rates in critically Ill trauma and surgical patients. *J Trauma.* (2010) 68:874–80. 10.1097/TA.0b013e3181d32271 20386282

[8] Kramme K Sarraf P Munene G. Prophylactic enoxaparin adjusted by anti-factor Xa peak levels compared with recommended thromboprophylaxis and rates of clinically evident venous thromboembolism in surgical oncology patients. *J Am Coll Surg.* (2020) 230:314–21. 10.1016/j.jamcollsurg.2019.11.012 31843692

[9] Ko A Harada MY Barmparas G Chung K Mason R Yim DA Association between enoxaparin dosage adjusted by anti-factor Xa trough level and clinically evident venous thromboembolism after trauma. *JAMA Surg.* (2016) 151:1006–13. 10.1001/jamasurg.2016.1662 27383732

[10] Rakhra S Martin E-L Fitzgerald M Udy A. The ATLANTIC study: anti-Xa level assessment in trauma intensive care. *Injury.* (2020) 51:10–4. 10.1016/j.injury.2019.10.066 31679829

[11] Vlot EA Van den Dool EJ Hackeng CM Sohne M Noordzij PG Van Dongen EPA. Anti Xa activity after high dose LMWH thrombosis prophylaxis in covid 19 patients at the intensive care unit. *Thromb Res.* (2020) 196:1–3. 10.1016/j.thromres.2020.07.035 32805623 PMC7374121

[12] Bethea A Adams E Lucente FC Samanta D Chumbe JT. Improving pharmacologic prevention of VTE in trauma: IMPACT-IT QI project. *Am Surg.* (2018) 84:1097–104. 10.1177/000313481808400672 29981655 PMC6368171

[13] Kopelman TR Walters JW Bogert JN Basharat U Pieri PG Davis KM Goal directed enoxaparin dosing provides superior chemoprophylaxis against deep vein thrombosis. *Injury.* (2017) 48:1088–92. 10.1016/j.injury.2016.10.039 28108019

[14] Lewis TC Cortes J Altshuler D Papadopoulos J. Venous thromboembolism prophylaxis: a narrative review with a focus on the high-risk critically Ill patient. *J Intens Care Med.* (2019) 34:877–88. 10.1177/0885066618796486 30165770

[15] Levine MN Planes A Hirsh J Goodyear M Vochelle N Gent M. The relationship between anti-factor Xa level and clinical outcome in patients receiving enoxaparine low molecular weight heparin to prevent deep vein thrombosis after hip replacement. *Thromb Haemost.* (1989) 62:940–4. 10.1055/s-0038-1651032 2556813

[16] Bara L Planes A Samama M-M. Occurrence of thrombosis and haemorrhage, relationship with anti-Xa, anti-IIa activities, and D-dimer plasma levels in patients receiving a low molecular weight heparin, enoxaparin or tinzaparin, to prevent deep vein thrombosis after hip surgery. *Br J Haematol.* (1999) 104:230–40. 10.1046/j.1365-2141.1999.01153.x 10050702

[17] Vahtera A Vaara S Pettilä V Kuitunen A. Plasma anti-FXa level as a surrogate marker of the adequacy of thromboprophylaxis in critically ill patients: a systematic review. *Thromb Res.* (2016) 139:10–6. 10.1016/j.thromres.2015.12.016 26916290

[18] Van PY Cho SD Underwood SJ Morris MS Watters JM Schreiber MA. Thrombelastography versus antiFactor Xa levels in the assessment of prophylactic-dose enoxaparin in critically ill patients. *J Trauma.* (2009) 66:1509–15; discussion 1515–17. 10.1097/TA.0b013e3181a51e33 19509608

[19] Hornung P Khairoun M Dekker FW Kaasjager KAH Huisman A Jakulj L Dosage reduction of low weight heparin in patients with renal dysfunction: effects on anti-Xa levels and clinical outcomes. *PLoS One.* (2020) 15:e0239222. 10.1371/journal.pone.0239222 33001983 PMC7529211

[20] Andersen NG Mowinckel M-C Sunde K Sandset PM Beitland S. Utility of coagulation analyses to assess thromboprophylaxis with dalteparin in intensive care unit patients. *Acta Anaesthesiol Scand.* (2021) 65:489–98. 10.1111/aas.13748 33205407

[21] Farrar JE Droege ME Philpott CD Mueller EW Ernst NE Makley AT Impact of weight on anti-Xa attainment in high-risk trauma patients on enoxaparin chemoprophylaxis. *J Surg Res.* (2021) 264:425–34. 10.1016/j.jss.2021.03.020 33848842

[22] Eck RJ Hulshof L Wiersema R Thio CHL Hiemstra B van den Oever NCG Incidence, prognostic factors, and outcomes of venous thromboembolism in critically ill patients: data from two prospective cohort studies. *Crit Care.* (2021) 25:27. 10.1186/s13054-021-03457-0 33436012 PMC7801861

[23] The Protect Investigators for the Canadian Critical Care Trials Group and the Australian and New Zealand Intensive Care Society Clinical Trials Group Cook D Meade M Guyatt G Walter S Heels-Ansdell D Dalteparin versus unfractionated heparin in critically Ill patients. *N Engl J Med.* (2011) 364:1305–14. 10.1056/NEJMoa1014475 21417952

[24] Sutzko DC Georgoff PE Obi AT Healy MA Osborne NH. The association of venous thromboembolism chemoprophylaxis timing on venous thromboembolism after major vascular surgery. *J Vasc Surg.* (2018) 67:262–71.e1. 10.1016/j.jvs.2017.06.087 28870681 PMC5741504

[25] Louis SG Sato M Geraci T Anderson R Cho SD Van PY Correlation of missed doses of enoxaparin with increased incidence of deep vein thrombosis in trauma and general surgery patients. *JAMA Surg.* (2014) 149:365. 10.1001/jamasurg.2013.3963 24577627

[26] Nathens AB McMurray MK Cuschieri J Durr EA Moore EE Bankey PE The practice of venous thromboembolism prophylaxis in the major trauma patient. *J Trauma.* (2007) 62:557–63. 10.1097/TA.0b013e318031b5f5 17414328

[27] Gratz J Wiegele M Maleczek M Herkner H Schöchl H Chwala E Risk of clinically relevant venous thromboembolism in critically Ill patients with COVID-19: a systematic review and meta-analysis. *Front Med.* (2021) 8:647917. 10.3389/fmed.2021.647917 33768106 PMC7985162

[28] Ball J. Venous thromboembolism in critically Ill patients requires significant reconsideration. *Crit Care Med.* (2020) 48:934–5. 10.1097/CCM.0000000000004324 32433086

