# Supplementary material for: Anti-factor Xa Activity Is Not Associated With Venous Thromboembolism in Critically Ill Patients Receiving Enoxaparin for Thromboprophylaxis: A Retrospective Observational Study
Source: Front Med (Lausanne). 2022 Apr 29;9:888451. doi: 10.3389/fmed.2022.888451 (PMC9103187; doi:10.3389/fmed.2022.888451)
Supplement: Supplementary file 1 [file Data_Sheet_1.DOCX]

Supplementary Material

# Search strategy, keywords and detailed results to identify patients who developed venous thromboembolism

We searched ICU admission and discharge notes as well as daily clinical progress notes of intensive care staff using regular expressions matching various keywords indicating the presence of venous thrombosis or pulmonary embolism. Regular expressions used are shown in Table 1. Note that mostly german terms are matched.

| Spiral(-\| )+\w*(?!tubus) |
| --- |
| \w*(?<!Luft\|Fruchtwasser)embol(?!isation)\w+ |
| (?<!LVO)TVT\|DVT\|VTE |
| (?!Thrombozyt\|Thromb\w*penie)Th?r?ombu\w* |
| \w*(?<!wund\|bauch\w*\|stoma\|naht\|vaginal?\|hern\w*\|abdom\w*\|zwerch\w*\|bruch\w*\|teil\|direkt\|thorax\|sekundär\|primär\|sternum\|brustbein\|taschen)verschl[uü](?:ss\|ß\|s)\w*\b |
| \w*gerinn(?!ung)\w* |
| \w*th?r?ombosi\w* |
| embolism |
| Spirale |
| \w*-PE\b |
| \bPEs?\b |
| Cavaschirm |
| Thrombe\w* |
| Thorax(?:-\| )(?:CT\|Computertomographie) |
| Thrombose(?!prophylaxe)\w* |
| verschlossen |
| Table 1: Regular expressions matching venous thrombombolism or pulmonary embolism. |

1,493,123 notes from 2510 patients were exported from the patient management data system. The automated search yielded 3965 matches, all of which were checked by one investigator (CD). Further patient documentation and radiological reports were checked as needed and diagnosed VTE were recorded. A total of 380 thrombotic events were identified, consisting of newly diagnosed VTE, prior VTE in patient history, VTE at admission, chronic thromboembolic pulmonary hypertension, portal vein thrombosis, cardiac thrombosis, arterial thrombosis, stroke and veinous thrombosis associated with extracorporeal membrane oxygenation.

# Details on patients with venous thromboembolism

| **VTE** | **Age**  **(years)** | **Sex** | **Died** | **LOS**  **(days)** | **Diagnosis** | **SAPS** | **Last measured Anti-factor Xa** | | | | | |
| --- | --- | --- | --- | --- | --- | --- | --- | --- | --- | --- | --- | --- |
|  |  |  |  |  |  |  | **Peak** | | **12 hour trough** | | **24 hour trough** | |
|  |  |  |  |  |  |  | **Value**  **(IU/mL)** | **Interval***  **(days)** | **Value**  **(IU/mL)** | **Interval***  **(days)** | **Value**  **(IU/mL)** | **Interval***  **(days)** |
| DVT | 32 | F | Yes | 145 | Stroke | 88 |  |  |  |  | 0 | 15 |
| DVT | 64 | F | No | 201 | Acute Abdomen | 77 | 0.21 | 34 | 0.36 | 36 |  |  |
| DVT | 78 | M | No | 44 | Aneurysm of iliac artery | 100 | 0.00 | 1 |  |  |  |  |
| DVT | 35 | M | Yes | 56 | ARDS | 58 |  |  | 0.17 | 1 |  |  |
| DVT | 59 | F | Yes | 144 | Thoracal discitis | 76 |  |  | 0.22 | 23 |  |  |
| PE | 43 | F | No | 27 | Esophago-pulmonary fistula | 35 |  |  |  |  | 0 | 4 |
| PE | 66 | F | No | 9 | Endometrial carcinoma | 58 |  |  | 0.14 | 1 |  |  |
| PE | 52 | F | No | 92 | Rectal carcinoma | 58 |  |  | 0.00 | 1 |  |  |
| PE | 74 | F | No | 7 | Acute kidney injury | 53 | 0.33 | 1 |  |  |  |  |
| PE | 80 | M | No | 49 | Abdominal aortic aneurysm | 40 | 0.30 | 26 |  |  |  |  |
| PE | 45 | M | No | 48 | Lung transplant | 40 |  |  | 0.21 | 1 |  |  |
| PE | 27 | M | No | 25 | Combined liver and kidney transplant | 58 |  |  | 0.00 | 1 |  |  |
| PE | 84 | M | No | 12 | Abdominal aortic aneurysm | 90 |  |  | 0.13 | 0 |  |  |
| PE | 61 | M | No | 8 | Sarkoma | 50 | 0.00 | 0 |  |  |  |  |
| UEDVT | 23 | M | Yes | 43 | Burn injury | 64 | 0.00 | 37 |  |  |  |  |
| UEDVT | 76 | F | Yes | 79 | Sepsis | 86 | 0.27 | 47 |  |  | 0 | 56 |
| UEDVT | 61 | M | Yes | 89 | Liver transplant | 76 |  |  | 0.00 | 1 | 0 | 4 |
| UEDVT | 71 | M | No | 68 | Sepsis | 98 | 0.30 | 17 |  |  |  |  |
| UEDVT | 36 | F | No | 4 | Lung transplant | 46 | 0.33 | 1 |  |  |  |  |
| VTE: venous thromboembolism, DVT: deep venous thrombosis, PE: pulmonary embolism, UEDVT: upper extremity deep venous thrombosis, LOS: length of stay, SAPS: severe acute physiology score (at admission), IU: international units, M: male, F: female * interval given in days before diagnosis of VTE | | | | | | | | | | | | |

# Anti-factor Xa activity stratified by illness severity

| **SAPS Quarter** | **Peak** | | | **12-hour trough** | | | **24-hour trough** | | |
| --- | --- | --- | --- | --- | --- | --- | --- | --- | --- |
|  | **No VTE** | **VTE** | **p-value** | **No VTE** | **VTE** | **p-value** | **No VTE** | **VTE** | **p-value** |
| 35 (29-38) | 0.21 (0.15-0.27) | 0.33 (0.33-0.33) | 0.227 | 0.1 (0-0.15) | 0.26 (0.26-0.26) | 0.116 | 0 (0-0) | 0 (0-0) | 0.682 |
| 46 (43-49) | 0.22 (0.15-0.32) | 0.16 (0.08-0.25) | 0.666 | 0.1 (0-0.16) | - | - | 0 (0-0) | - | - |
| 58 (56-62) | 0.20 (0.12-0.32) | 0.22 (0.11-0.33) | >0.999 | 0.1 (0-0.15) | 0 (0-0.03) | 0.197 | 0 (0-0) | - | - |
| 74 (69-81) | 0.21 (0.13-0.32) | 0.29 (0.21-0.31) | 0.687 | 0.12 (0-0.18) | 0.2 (0.1-0.28) | 0.343 | 0 (0-0.11) | 0 (0-0) | 0.282 |
| SAPS Missing | 0.23 (0.14-0.29) | - | - | 0.04 (0-0.18) | - | - | 0 (0-0) | - | - |
| Median, minimum and maximum anti-factor Xa activities were calculated for each patient and summarised using median (interquartile range). p-values were obtained using Wilcoxon rank sum tests. | | | | | | | | | |
